# Exploration of the Specific Pathology of HXMM Tablet Against Retinal Injury Based on Drug Attack Model to Network Robustness

**DOI:** 10.3389/fphar.2022.826535

**Published:** 2022-03-25

**Authors:** Yujie Xi, Yan Miao, Rui Zhou, Maolin Wang, Fangbo Zhang, Yu Li, Yi Zhang, Hongjun Yang, Feifei Guo

**Affiliations:** ^1^ Institute of Chinese Materia Medica, China Academy of Chinese Medical Sciences, Beijing, China; ^2^ Chinese Medicine Research Institute, Tianjin University of Traditional Chinese Medicine, Tianjin, China; ^3^ Department of Pharmacology, School of Basic Medical Sciences, Xi’an Jiaotong University Health Science Center, Xi’an, China; ^4^ College of Traditional Chinese Medicine, Guangzhou University of Chinese Medicine, Guangzhou, China; ^5^ Beijing Key Laboratory of Traditional Chinese Medicine Basic Research on Prevention and Treatment for Major Diseases, Experimental Research Center, China Academy of Chinese Medical Sciences, Beijing, China

**Keywords:** he xue ming mu tablet, age-related macular degeneration, diabetic retinopathy, oxidative stress, inflammation, network robustness, drug attack

## Abstract

Retinal degenerative diseases are related to retinal injury because of the activation of the complement cascade, oxidative stress-induced cell death mechanisms, dysfunctional mitochondria, chronic neuroinflammation, and production of the vascular endothelial growth factor. Anti-VEGF therapy demonstrates remarkable clinical effects and benefits in retinal degenerative disease patients. Hence, new drug development is necessary to treat patients with severe visual loss. He xue ming mu (HXMM) tablet is a CFDA-approved traditional Chinese medicine (TCM) for retinal degenerative diseases, which can alleviate the symptoms of age-related macular degeneration (AMD) and diabetic retinopathy (DR) alone or in combination with anti-VEGF agents. To elucidate the mechanisms of HXMM, a quantitative evaluation algorithm for the prediction of the effect of multi-target drugs on the disturbance of the disease network has been used for exploring the specific pathology of HXMM and TCM precision positioning. Compared with anti-VEGF agents, the drug disturbance of HXMM on the functional subnetwork shows that HXMM reduces the network robustness on the oxidative stress subnetwork and inflammatory subnetwork to exhibit the anti-oxidation and anti-inflammation activity. HXMM provides better protection to ARPE-19 cells against retinal injury after H_2_O_2_ treatment. HXMM can elevate GSH and reduce LDH levels to exhibit antioxidant activity and suppress the expression of IL-6 and TNF-α for anti-inflammatory activity, which is different from the anti-VEGF agent with strong anti-VEGF activity. The experimental result confirmed the accuracy of the computational prediction. The combination of bioinformatics prediction based on the drug attack on network robustness and experimental validation provides a new strategy for precision application of TCM.

## 1 Introduction

The eye, as a window connecting humans and the external world, gives humans the ability to distinguish external things and colors. The retina in the eye is a complex neural structure that integrates visual information. Damage to retina often causes irreversible damage to vision, including chronic progressive visual field loss and retinopathy pigmentosa, even complete blindness. A nationwide online survey found that blindness ranked as the third major fear (after cancer and heart disease) (Scott et al., 2016). Degeneration of the retinal pigment epithelium (RPE) is the main characteristic of retinal degenerative diseases in the elderly population. But the underlying disease mechanism has not yet been identified, mainly due to the multifactorial nature of this disease. Retinal degenerative diseases can cause blindness and bring a huge disease burden to patients. In particular, age-related macular degeneration (AMD) and diabetic retinopathy (DR) are the most common fundus vascular diseases in the clinic. Because of their high incidence, serious disability, and representativeness, we take these two diseases as examples to discuss. Here, we review some of the commonly proposed degeneration pathways of RPE cells and their roles in the pathogenesis of AMD and DR, including activation of the complement cascade, oxidative stress-induced cell death mechanisms, dysfunctional mitochondria, chronic neuroinflammation mediated by microglial cells, and production of the vascular endothelial growth factor (VEGF) (Cheung et al., 2010; Barben et al., 2018; Massengill et al., 2018; Somasundaran et al., 2020). In addition, accumulating evidence indicates that neurodegeneration, neuroinflammation, and renin–angiotensin system (RAS) activation also played important roles in DR development. Furthermore, the aberrant production of mitochondria-derived reactive oxygen species and endoplasmic reticulum (ER) stress are also involved in the pathogenesis of DR (Cheung et al., 2010). Anti-VEGF therapy demonstrated remarkable clinical benefits in AMD and DR patients; however, the majority of patients failed to achieve significant visual improvement, which was associated with the involvement of other molecular pathways than VEGF. In addition, anti-angiogenic therapy, anti-inflammatory therapy, and laser treatment were applied in retinal degenerative disease treatment. Yet, it remains difficult for patients with severe visual loss to achieve reading or driving vision with currently available therapeutics (Wang and Lo, 2018).

It is well known that traditional Chinese medicine (TCM) has a polypharmacological effect. It exerts pharmacological activity through multi-target effects in the prevention and treatment of complex diseases. Based on multiple targets, TCM can produce a synergistic effect in the disease network, making the total effect greater than the sum of the individual effects and ultimately achieving the best therapeutic effect. This coincides with the traditional view that medicine has its own specialties, and it has the magical effect of being in a group (Kuenzi et al., 2017). He xue ming mu (HXMM) tablet is a China Food and Drug Administration (CFDA)-approved TCM for retinal degenerative diseases (FD), which consists of 19 herbs, including *Typha domingensis* Pers (*Typhaceae*) (pu huang, 75 g, Neimenggu), *Salvia miltiorrhiza* Bunge (*Lamiaceae*) (dan shen, 75 g, Shandong), *Rehmannia glutinosa* (Gaertn.) DC (*Orobanchaceae*) (di huang, 60 g, Henan), *Eclipta prostrata* (L.) L (*Asteraceae*) (mo han lian, 60g, Henan), *Chrysanthemum × morifolium* (Ramat.) Hemsl (*Asteraceae*) (ju hua, 50 g, Anhui), *Scutellaria baicalensis* Georgi (*Lamiaceae*) (huang qin, 45 g, Shanxi), *Senna tora* (L.) Roxb (*Fabaceae*) (jue ming zi, 45 g, Henan), *Plantago ovata* Forssk (*Plantaginaceae*) (che qian zi, 45 g, Jiangxi), *Leonurus japonicus* Houtt (*Lamiaceae*) (chong wei zi, 45 g, Henan), *Ligustrum lucidum* W.T. Aiton (*Oleaceae*) (nu zhen zi, 45 g, Shanxi), *Prunella vulgaris* L. (*Lamiaceae*) (xia ku cao, 45 g, Anhui), *Gentiana scabra* Bunge (Gentianaceae) (long dan, 45 g, Liaoning), *Curcuma aromatica* Salisb (Zingiberaceae) (yu jin, 30 g, Guangxi), *Equisetum hyemale* L. (*Equisetaceae*) (mu zei, 45 g, Liaoning), *Paeonia lactiflora* Pall (*Paeoniaceae*) (chi shao, 30 g, Neimenggu), *Paeonia × suffruticosa* Andrews (*Paeoniaceae*) (mu dan pi, 30 g, Anhui), *Crataegus pinnatifida* Bunge (*Rosaceae*) (shanzha, 30 g, Shandong), *Angelica sinensis* (Oliv.) Diels (*Apiaceae*) (dang gui, 30 g, Gansu), *Conioselinum anthriscoides* “Chuanxiong” (*Apiaceae*) (Chuanxiong 10 g, Sichuan). This basic information is recorded in the Chinese pharmacopoeia (USP, 2015). HXMM can alleviate the symptoms of AMD and DR alone or in combination with anti-VEGF agents (Li et al., 2022), without having a thoroughly elucidated mechanism. *Typha domingensis Pers* (*Typhaceae*) (pu huang), *Salvia miltiorrhiza* Bunge (*Lamiaceae*) (dan shen), *Curcuma aromatica* Salisb (Zingiberaceae) (yu jin), and Conioselinum anthriscoides “Chuanxiong” (*Apiaceae*) (Chuanxiong) were reported with anti-inflammatory (Varpe et al., 2012; Ma S. et al., 2016; Pintatum et al., 2020; Shi et al., 2020), anti-oxidant (Liu et al., 2008; Pintatum et al., 2020; Shi et al., 2020), and anti-VEGF activities (Qian et al., 2011). Scutellaria baicalensis (huang qin) has anti-inflammatory and immunostimulatory activities (Qi et al., 2017). *Salvia miltiorrhiza* (dan shen) and *Ligusticum chuanxiong* (chuan xiong) also can prevent blood coagulation (Cao et al., 2015; Yang et al., 2020). With a combination of these herbs, HXMM may have anti-inflammatory, anti-oxidant, anti-VEGF, and anticoagulants activities, which are the key pathological processes of retinal degenerative diseases (Gao et al., 2020; Chen et al., 2022).

However, it is difficult to elucidate the mechanisms of HXMM in treating retinal degenerative diseases because this involves connecting a multi-component drug with multi-target to multifactorial diseases. Based on the “multi-component and multi-target” principle, network robustness methods can be learned from network sciences to identify drug positioning for disease. The complex disease can be described by networks, in which gene interactions in specific disease conditions are represented by vertices and edges between vertices. In order to associate drugs with diseases and disease functional subnetworks, we evaluate the perturbation of multi-target drugs on the disease network, from the multi-target effect to the robustness of the whole network instead of a single target. Health systems are generally robust against drug attacks, but the disease network can be fragile facing with perturbations (Kitano, 2007). Drug attacks with a strong reduction of the robustness of the disease network suggest that this drug may be more effective for this disease or pathological process. The quantitative evaluation algorithm for the prediction of the effect of multi-target drugs on the disturbance of the disease network had been used for drug positioning on hypertension nephropathy (Guo et al., 2019) and drug discovery against COVID-19 (Guo et al., 2020), which demonstrate the potential of drug precision positioning for multi-target drugs.

In this study, a large complex network model of AMD and DR was divided into multiple functional subnetworks (including oxidative stress subnetwork, inflammatory subnetwork, angiogenesis subnetwork, and so on) to evaluate the robustness of different functional disease subnetworks after multi-target drug attack, which reflect the therapeutic characteristics of the drugs. As the main clinical drug with high-level evidence used to treat AMD and DR, anti-VEGF agents had a stronger disturbance to the disease network than other types of adjuvant therapy (including growth factors, corticosteroids, anticoagulants, angiotensin II receptor blockers, and so on), which indicates that this algorithm has a good prediction performance for drug positioning. Compared with anti-VEGF agents, the drug disturbance of HXMM on the functional subnetwork shows that HXMM reduces the network robustness on the oxidative stress subnetwork and inflammatory subnetwork, which indicates that HXMM may have anti-oxidation and anti-inflammatory activities. Experimental validation shows that HXMM provides better protection of acute retinal pigment epithelial (ARPE-19) cells against retinal injury after H_2_O_2_ treatment by elevating glutathione (GSH) and decreasing lactate dehydrogenase (LDH) levels to exhibit anti-oxidant activity and suppressing the expression of interleukin-6 (IL-6) and tumor necrosis factor-α (TNF-α) for anti-inflammatory activity. HXMM is different from the anti-VEGF agent with strong anti-oxidation activity, with equivalent ability for anti-inflammation and anti-VEGF. The combination of bioinformatics prediction based on the drug attack on network robustness and experimental validation provides a new strategy for precision application of drugs.

## 2 Materials and Methods

### 2.1 Preparation of He Xue Ming Mu Tablet Everted Gut Sac Liquid

Adult male Sprague–Dawley (SD) rats weighing 230–250 g were provided by SiPeiFu(Beijing) Biological Technology Co., Ltd., China (Certificate NO. SCXK (Jing) 2019-0010). The experiment was approved by the Committee on Animal Care and Use of Beijing, China. Under the condition of fasting for at least 12 h before the experiment, rats were executed by cervical dislocation, and then the intestine was rapidly removed and divided into four segments (duodenum, jejunum, ileum, and colon). Each of them were washed with ice-cold Tyrode buffer (distilled water containing 0.05 g/L NaH_2_PO_4_, 0.10 g/L MgCl_2_, 0.20 g/L CaCl_2_, 0.28 g/L KCl, 1.00 g/L NaHCO_3_, 1.00 g/L glucose, 8.00 g/L NaCl, pH 7 0.4). Every segment was everted gently and one end was ligated with silk thread. Tyrode buffer (2 ml) was added to fill it up and then tied firmly to the other end. Finally, the sac was transferred to a Magnus bath containing 20 ml HXMM liquid with oxygenated media (95%O_2_/5%CO_2_) at 37°C. The samples can be done after 2 h intervals and treated by 0.22 μm millipore filtration to store (Alam et al., 2012; Zhang et al., 2013). The original HXMM intestinal absorption liquid was 200 mg/ml (crude drug). The animal study was reviewed and approved by the Institute of Chinese Materia Medica, China Academy of Chinese Medical Sciences (Beijing, China).

### 2.2 Identification of Active Compounds of He Xue Ming Mu Tablet by HPLC-MS

HXMM intestinal absorption liquid was analyzed by the UHPLC-Q-Orbitrap HRMS system of Ultimate 3000 HPLC (Dionex, United States of America) and Thermo Q Exactive Plus HRMS (Thermo Fisher Scientific, United States of America). The analysis was conducted by Waters ACQUITY UPLC HSS T3 C18 (2.1 mm × 100 mm, 1.8 μm, Waters Corporation, United States), while the mobile phase consisted of 0.1% formic acid acetonitrile (A) formic acid water (B) which was used for gradient elution, with the flow rate of 0.2 ml/min. The optimized gradient program was as follows: from 0 to 10 min (100%B); 10–20 min (100–70% B); 10–25 min (70–60% B); 25–30 min (60–50% B); 30–40 min (50–30% B); 40–45 min (30–0% B); 45–60 min (0% B); 60–60.1 min (0%–100%B); back to 100% B within 10 min and the column temperature was maintained at 35°C. In this study, both positive and negative ion modes with a heated electrospray ionization (HESI) source were designed for mass detection. Some parameters were maintained as follows: ion spray voltage of + 3200–3000 V; with a resolution of 70,000 MS, and 17,500 MS/MS. The quasi-molecular ion peaks, fragment ion information, and retention time provided by the UHPLC-Q-Orbitrap system in the HXMM group and blank group were analyzed and compared. The unknown compounds were identified by Compound Discover 3.2 software to search the mzcloud and mzVault databases, including peak matching, peak alignment, noise filtering, normalization, etc.

### 2.3 Drug Target Acquisition and Processing

The intestinal-absorbable compounds of 19 herbs of HXMM were identified based on HPLC-MS, whereas the targets of these compounds were predicted by BATMAN-TCM (Liu et al., 2016). The drug targets were selected with a high confidence score (score cutoff greater than 20) as potential targets of HXMM (Liu et al., 2016). The targets of FDA-approved drugs were predicted based on the LINCS (Library of Integrated Network-based Cellular Signatures) program launched by the National Institutes of Health. We used the L1000 gene expression data set to analyze the gene expression data profile and ensure the changes of transcriptome genes after 6 and 24 h of drug intervention in 10 selected cell types. Pearson correlation analysis was applied to discover gene expression changes that are significantly related to the time of drug administration. After integration and analysis, the top 1000 genes with correlation coefficients were defined as potential (direct or indirect) target points of FDA-approved drugs (Haider, 2010; Öztürk et al., 2016).

### 2.4 Construction of Disease Network and Functional Subnetwork of AMD and DR

Disease genes related to AMD (C0242383) and DR (C0011884) were downloaded *via* the DisGeNET (Piñero et al., 2017) (http://www.disgenet.org) database on 8 Mar 2021. We used AMD and DR as keywords with the website to match the appropriate disease and then got the genes associated with these two diseases at once. The data of gene-disease associations can be sorted by GDA (gene-disease association) score, and the cutoff was more than 0.01. The disease network was constructed by protein-protein interactions (PPI) of AMD and DR-related genes separately, whose PPI confidence score was greater than 0.4 from the STRING (von Mering et al., 2005; Szklarczyk et al., 2017) (version 10). The functional enrichment analysis of Gene Ontology (GO) function items involved in the disease genes was realized by Metascape software (Zhou et al., 2019). The cutoff of FDR of GO enrichment is less than 0.01, and the FDR is calculated by the accumulative hypergeometric distribution and corrected by multiple testing corrections. Those enriched terms were clustered into several different subclusters according to hierarchical clustering based on the similarity distance matrix of GO terms by Metascape (https://metascape.org). The disease genes involved in one subcluster were used to construct a functional subnetwork based on PPI interaction from STRING. For each functional subnetwork, if the protein interaction occurred in more than four subnetworks, the corresponding interaction was deleted to form a new subnetwork.

### 2.5 Quantitative Evaluation Algorithm for Prediction of Effect of Multi-Target Drugs on Disease Based on Network Robustness After Drug Attack

Drug attack on network robustness was used as a quantitative evaluation algorithm for the prediction of the effect of multi-target drugs on disease. Specifically, the disturbing effect of drugs on diseases is simulated by deleting the drug targets of disease networks. The purpose of a drug attack on the disease network is to maximize the damage to the structure of the disease network and to reduce the robustness of network. Here, the average shortest path length (ASPL), average degree (AD), degree centrality (DC), and closeness centrality (CC) were used as the four main indicators to evaluate the robustness of the network topology.

The average degree of the graph is simply the average number of degrees per node in the graph. The definition that is

$$AD_{net}=\frac{\sum_{v\in \;V}\deg\left(v\right)}{n},$$ (1)

where V is the set of nodes in G , deg(v) is the degree of node v , and n is the number of nodes in G .

The average shortest path length is one of the most robust measures of the network topology, which means that the average value of all the shortest path lengths in the network. A positive disturbance rate indicates that the drug has a strong ability to destroy the stability of the network. The ASPL of a whole network is

$$ASPL_{net}=\sum_{s,t\in V}\frac{d\left(s,t\right)}{n\left(n-1\right)},$$ (2)

where V is the set of nodes in G , d(s,t) is the shortest path from s to t , and n is the number of nodes in G .

The degree centrality is a centralization measure for the whole graph, which is calculated based on the centrality of the degrees of all nodes (Freeman, 1979; Borgatti, 2005; Saberi et al., 2021). The negative disturbance rate also indicates that the drug has a strong destructive ability to destroy the network. Let v* be the node with the highest degree in G. Correspondingly, the DC of the graph G is as follows:

$$DC_{net}=\frac{\sum_{v\in \;V}\left[\deg\left(v^{*}\right)-\deg\left(v\right)\right]}{\left(n-1\right)\left(n-2\right)},$$ (3)

where V is the set of nodes in G , deg(v) is the degree of node v , and n is the number of nodes in G .

Closeness centrality is another centralization measure for the whole graph, calculated from the closeness of all nodes (Vanderbilt Library Catalog, 2022) Let $v^{\#}$ be the node with highest closeness in G . Correspondingly, the closeness centrality of the graph G is as follows:

$$CC_{net}=\frac{\sum_{v\in G}\left[\text{clo}\left(v^{\#}\right)-\text{clo}\left(v\right)\right]}{\frac{\left(n-1\right)\left(n-2\right)}{2n-3}},$$ (4)

where V is the set of nodes in G , clo(v) is the degree of node v , and n is the number of nodes in G .

By comparing the changes in the network robustness topology before and after the drug attack, the robustness index (RI) is used to evaluate the intervention effect of drugs on the disease.

$$\text{Robustness Index}\left(\text{RI}\right)=\frac{Topological\;Feature_{after\;attack}-Topological\;Feature_{before\;attack}}{Topological\;Feature_{before\;attack}}$$ (5)

Since most of the drug targets belong to small sample data with unknown overall distribution, the permutation test is often used in the significance investigation. The essence of the disease network in null distribution is to generate random characteristics of the network topology after retaining the number of real network nodes and edges (Baldassano and Bassett, 2016). The null distribution generated by the random network can be used as a standard to compare the drug effects of the real disease network. Comparing the null distribution generated by 100 random networks with 100 random robustness indices after the same drug attack, we can objectively find that the real disturbance rate can make significant contributions to evaluate drug interference in a real disease network. Even to investigate the overall distribution of the disturbance rate and the network robustness between the real network and the random network it is important. And a normalized **RI** was generated as the z-score of RI of the real network compared to the null distribution. $\bar{\mathrm{RI}}$ is the mean of random RI in null distribution, and ***S*** is the standard deviation of random **RI** in null distribution (Guo et al., 2019).

$$\text{Normalized RI}=\frac{RI_{real}-{\bar{RI}}_{random}}{S_{random}}$$ (6)

For the RI of ASPL, the smaller the value, the more stable is the network. For drug RI of AD, DC, and CC, the bigger the value, the more stable is the disease network after the drug attack. So the calculation formula for the total score of robustness index of the disease network after the drug attack was as followed:

$$\text{Total Score}={\text{Normalized RI}}_{\text{ASPL}}-{\text{Normalized RI}}_{\text{AD}}-{\text{Normalized RI}}_{\text{DC}}-{\text{Normalized RI}}_{\text{CC}}$$ (7)

### 2.6 H_2_O_2_ Induced Retinal Injury Model in ARPE-19 Cells

#### 2.6.1 ARPE-19 Cell Culture and Treatment

ARPE-19 was purchased from the American Type Culture Collection (ATCC, United States of America). The cells were cultured in an incubator with 1640 (Gibco, United States of America), 10% fetal bovine serum (FBS, GIBCO, United States of America), 1% penicillin, and streptomycin (Gibco, United States of America) at 37°C with 5% CO2 in a humidified atmosphere. The culture medium was changed every 2 days, and cells were passaged when cell density reached more than 80%.

#### 2.6.2 Establishment of Retinal Injury Model in ARPE-19 Cells

ARPE-19 cells were seeded in 96-well plates (2 × 10^4^/well) and developed in full medium for 24 h. Briefly, cells were treated with H_2_O_2_ at concentrations of 50, 60, 80, and 100 μM for 12 h, and then cell viability was measured with Cell Counting Kit-8 (CCK-8, Dongren Chemical Technology Co., Ltd.). That is 10 μL CCK-8 was added to each well and observed for 2–4 h. Then the absorbance was validated by a microplate reader (Molecular Devices, United States of America) in 490 nm wavelength. Next the cell viability was calculated and the half-inhibition concentration (IC_50_) was selected as an appropriate concentration of H_2_O_2_ for further study.

#### 2.6.3 Evaluation of Toxicity of Drugs in ARPE-19 Cells

ARPE-19 cells were seeded in 96-well plates (2 × 10^4^/well) and cultured in 1640 with 10% FBS overnight. The cells were treated with HXMM intestinal absorption fluid at concentrations of 16, 8, 4, 2, 1.3, 1, and 0.5 mg/ml for 4 h or bevacizumab as a positive control at concentrations of 0.5, 0.25, 0.125, and 0.0625 mg/ml for 4 h. The cell survival rate was evaluated using a CCK-8 assay.

#### 2.6.4 Evaluation of HXMM Protection of ARPE-19 Cells Against Retinal Injury

ARPE-19 cells were seeded in 96-well plates (2 × 10^4^/well) for 24 h as usual. ARPE-19 cells were pretreated with three concentrations of HXMM (4, 2, and 1.3 mg/ml) for 4 h and then incubated with 65 μm H_2_O_2_ for 12 h. After that, the cell viability was measured by CCK-8.

#### 2.6.5 Biochemical Analysis of ARPE-19 Cell Supernatant

ARPE-19 cells were preserved by 4, 2, and 1.3 mg/ml concentrations of HXMM and 0.5 mg/ml bevacizumab for 4 h, while H_2_O_2_ (65 μm) was replaced for 12 h. The reactions were started by collecting the supernatant in groups and centrifuging the samples for 20 min at 1000 × g at 4°C. LDH assay kit measurement can be used for oxidative damage. The GSH assay kit (A006-2–1) was purchased for the Nanjing Jiancheng Bioengineering Institute. In addition, the enzyme-linked immunosorbent assay (ELISA) (Peprotech, United States of America) was used to assess the expression of inflammatory cytokines with IL-6, TNF-α, and angiogenesis secretory factor with VEGFA. All the experiments were conducted strictly following the manufacturer’s protocol.

#### 2.6.6 Measurement of ARPE-19 Cell Lysate

After establishing the retinal injury model in ARPE-19 cells and removing the supernatant, we needed to wash the cell twice gently with phosphate-buffered saline (PBS). Cell scrapers were provided to collect samples. To further break down, we used ultrasonic waves several times and centrifuged for 10 min at 1,500 × g. After the removal of fragments, the supernatant can be utilized for the assay. GSH kit (A020-3) was provided by the Nanjing Jiancheng Bioengineering Institute. All detection activity was determined by the manuscript instructions.

#### 2.6.7 Statistical Analysis

Each experiment duplicates at least three times and the data should be presented by means ± standard deviations (SD). GraphPad Prism 8.3.0 was used for statistical analysis. The results of different groups were compared by the Student t-test, and *p* < 0.05 was regarded as statistical significance.

## 3 Results

### 3.1 Identification of Compounds for He Xue Ming Mu Tablet Intestinal Absorption Solution

Based on HPLC-MS mining, 301 compounds were discovered in crude powder, to make further efforts to determine its intestinal-absorbable compounds, 226 intestinal-absorbable compounds of HXMM can be screened by the method of the everted gut sac. Among which, 201 compounds were found in positive ion mode condition, while 159 compounds were found in the condition of negative ion mode. More detailed information about active compounds can be seen in Supplementary Table S1.

### 3.2 He Xue Ming Mu Tablet Attack Reduces the Robustness of AMD and DR Disease Network

Eight hundred and fifty-four potential targets of HXMM were predicted by BATMAN-TCM, then 685 disease targets related to AMD and 645 disease targets related to DR were detected. Two PPI networks were constructed from the STRING for AMD and DR separately. The AMD network was composed of 636 nodes and 4193 edges and the DR network was composed of 578 nodes and 4632 edges. More detailed information about two disease networks was provided in Supplementary Figure S1.

**FIGURE 1:**
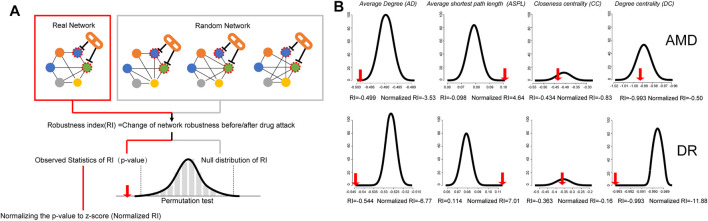
Schematic diagram of quantitative algorithm of the drug attack on network robustness **(A)** and permutation test of HXMM attack to AMD and DR network **(B)**. **(A)** Simulation of the disease network and the impact of drug attack on the robustness of the network. The schematic diagram simulates the calculation process of the impact of drug attack on the robustness of the disease network. In the same disease network, deleting different nodes will cause different changes in the network structure and robustness. Change of network topological features after drug attack was used as robustness index (RI) of networks. The null distribution of RI of 100 random networks was generated to maintain the same edge number of the true network and randomize interactions between nodes. The dotted area indicated the 95% confidence interval via the Permutation test. Compared with the null distribution, the statistical significance of RI of true network can be calculated and normalized RI can be corrected. We can normalize the *p*-value (observed statistics of RI) to z-score, which is (n-Normalized RI). By constructing a random network, simulating the overall distribution of the impact of deleted nodes on the robustness of the random network (permutation test), investigating the position of the drug on the real network disturbance in the overall distribution, calculating the corrected disturbance rate, and the intensity of drug disturbance on the real network can be evaluated objectively. **(B)** Permutation test of HXMM RIs of real network were shown. To evaluate the drug disturbance on the network from four topological features, including the AD, ASPL, CC, and BC. The red arrow pointed to the RI under the real network. The null distribution of RI of drug attack on the random network was shown as normal distribution curve. In AMD network, the normalized RI of AD, ASPL, DC and CC were -3.53, 4.64,-0.83 and 0.5, the corresponding figures of DR were -6.77, 7.01,-0.16.-11.88.

As shown in Figure 1A, normalized robustness index (normalized RI) of topological features was calculated based on the permutation test between the drug attack to real network and random network, which can evaluate the changes of network robustness after the drug attack. To comprehensively evaluate the strength of HXMM attack in AMD and DR disease network, real RI, and normalized RI of four topological features including average degree (AD), average shortest path length (ASPL), degree centrality (DC), and closeness centrality (CC) were calculated (Figure 1). The total score of normalized RI of four topological features was calculated, that AMD was 9.49, DR was 25.83, which implied that HXMM may have a certain intervention effect on these two diseases. Similarly, this method can be used to evaluate drug attacks to subnetwork of pathological processes of disease.

### 3.3 FDA-Approved Agents’ Attack on Subnetwork of Major Pathological Process of AMD and DR

**FIGURE 2:**
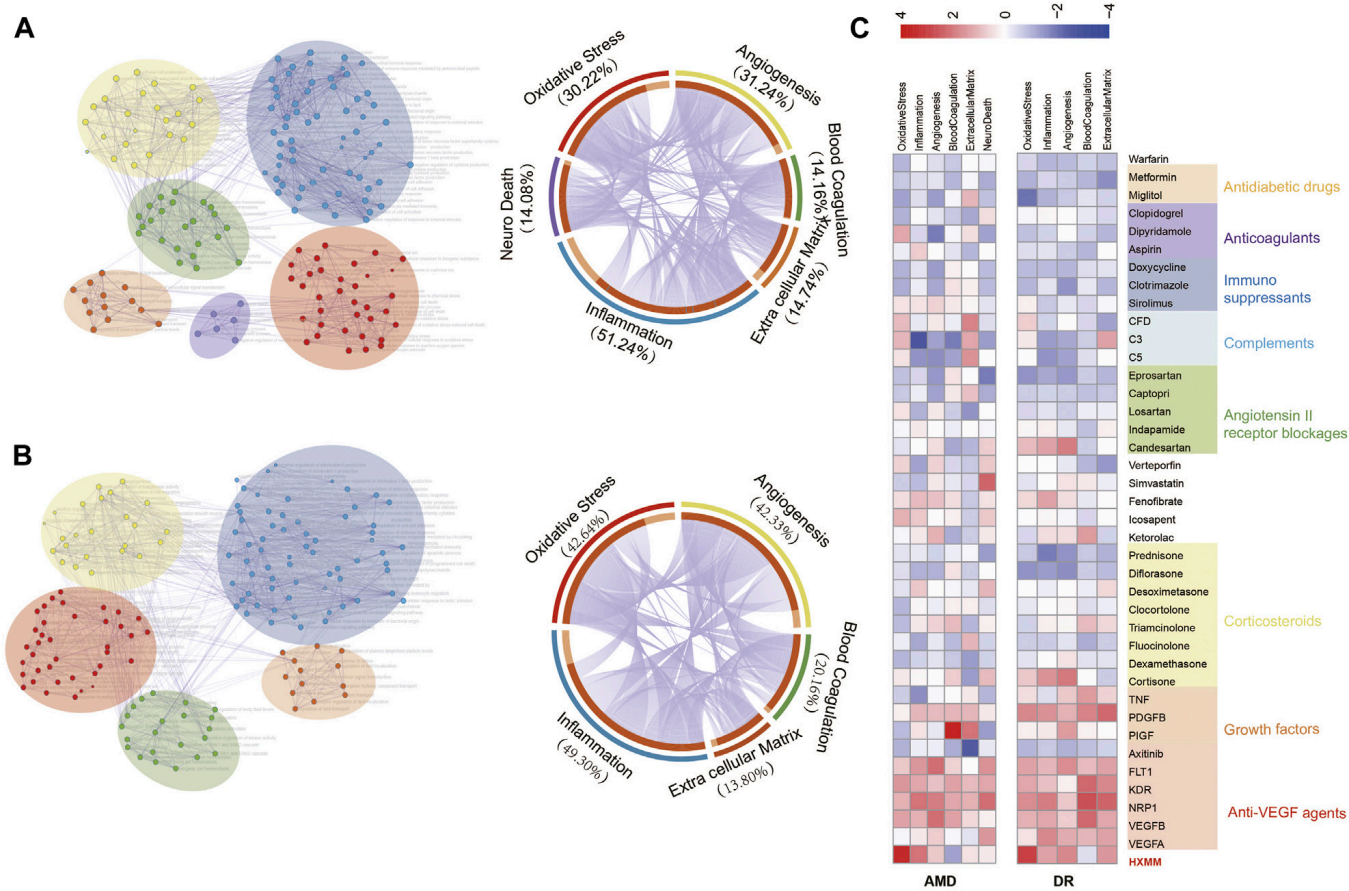
Major pathological process of AMD **(A)** and DR **(B)** and FDA-approved-drug disturbance on these pathological process subnetworks **(C)**. **(A,B)** Network of enriched biological process GO terms of AMD and DR (left), GO terms clustered in one group were marked with the same color (red—oxidative stress, blue—inflammation, yellow—angiogenesis, green—blood coagulation, organ—extracellular matrix, and purple—neuro death). Node size changes with the count of genes in this GO term. The circos plot of genes in GO terms cluster group shows the overlapping of major pathological processes. **(C)** FDA-approved-drug disturbance on these pathological process subnetworks. Anti-VEGF agents, growth factors, corticosteroids, angiotensin II receptor blockers, complements, immunosuppressants, anticoagulants, antidiabetic drugs were marked by blocks with different colors from red to gray.

Subsequently, enrichment analysis of the GO biological process was performed to explore the disease-related biological process Supplementary Figure S2. The results showed that disease gene of AMD participating in blood vessel morphogenesis, regulation of cytokine production, and response to wounding also played an important role in DR. To explore the specific process in AMD and DR, enriched GO terms of disease genes were clustered into several group clusters based on hierarchical clustering by Metascape (Figure 2A,B). Also the overlap of different group of AMD and DR were shown in two circus plots and the groups were renamed to represent the most GO terms in this group. In both AMD and DR, oxidative stress (30.22% in AMD and 42.64% in DR), inflammation (51.24% in AMD and 49.30% in DR), and angiogenesis (31.24% in AMD and 42.33% in DR) accounted for the three largest portions. The rest of the pathological process was making up that blood coagulation (14.16% in AMD and 20.16% in DR) and extracellular matrix (14.74% in AMD and 13.80% in DR) However, neuron death was unique to AMD (14.08%). The circus plots showed that many genes were overlapping between oxidative stress, inflammation, and angiogenesis which indicate different pathological processes may crosstalk with each other. In order to investigate the drug attack to different pathological processes of disease, six functional subnetworks for AMD and five for DR were constructed based on the PPI of genes in the GO terms for the same group. As a control, drug perturbance of 39 FDA-approved drugs was evaluated on the functional subnetwork of the diseases to verify the accuracy of the algorithm we developed. A heatmap of the total score of normalized RI for 39 drugs to six subnetworks in AMD and five subnetworks in DR were performed, revealing that the score of anti-VEGF agents, a clinical drug for AMD and DR with high-level evidence, were superior to any other drugs with middle or low-level evidence (Figure 2C). This result shows that our prediction method has a good prediction performance. Moreover, Figure 2C also showed that the total score of RI of HXMM was superior to 82.00% FDA-approved drugs in AMD, 87.00% FDA-approved drugs in DR.

### 3.4 He Xue Ming Mu Tablet and Its Constituent Herbs Attack on the Subnetwork of Major Pathological Processes of AMD and DR

**FIGURE 3:**
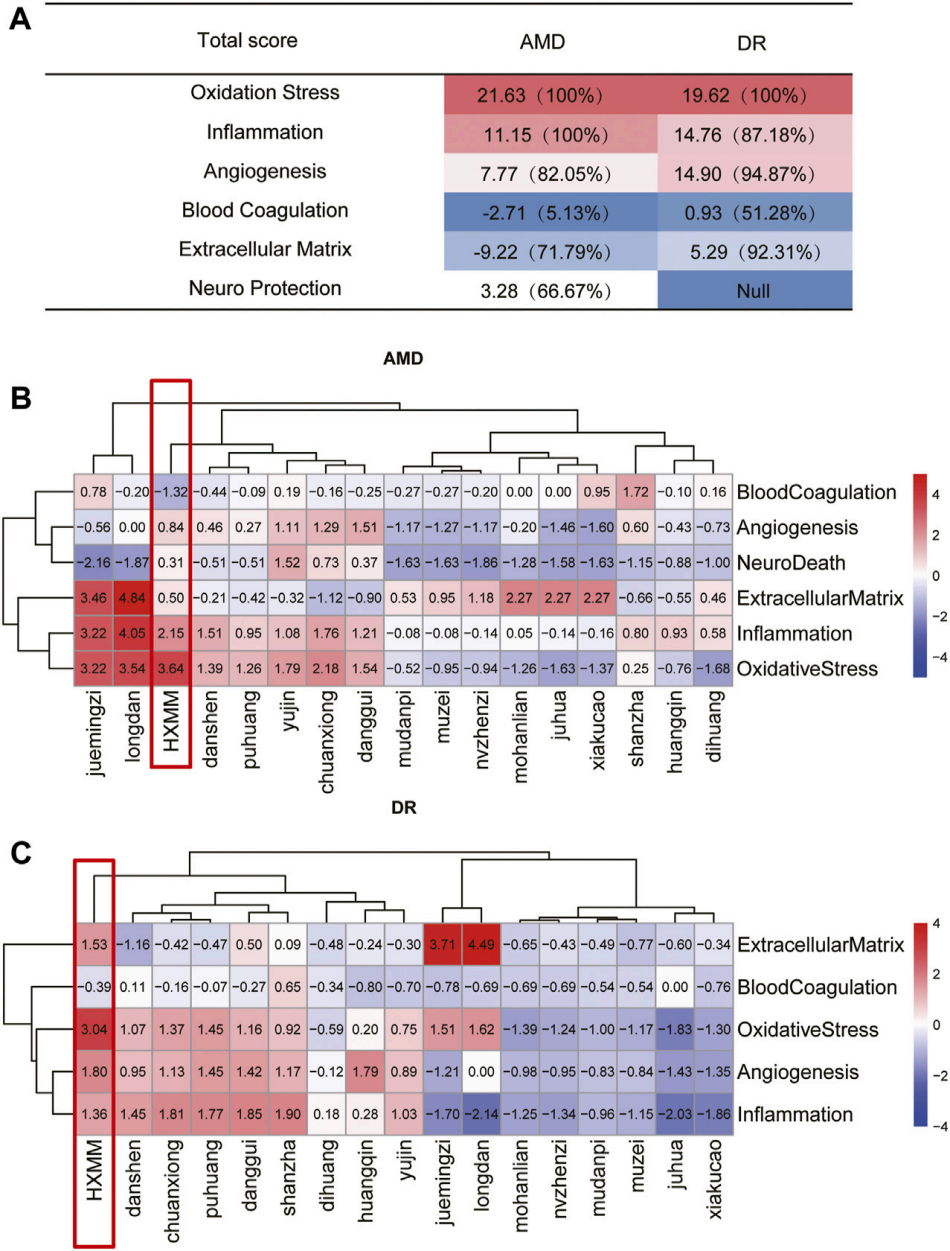
Evaluation of the Robustness of disease network disturbance after multi-target drug attack. **(A)** Distribution of total scores for different pathological processes in AMD and DR. The scores in brackets indicated the ratio of HXMM total scores greater than those FDA-approved drugs under the same pathological process. **(B)** Multi-target drug attack prediction on topological scores for AMD. **(C)** Multi-target drug attack prediction on topological scores for DR. The color depth changed with the increase of the score. The red was a positive number, which represented a stronger positive disturbing effect, and the blue was a negative number, which meant that it had a relatively weak disturbing ability. Among them, HXMM had shown better scores in multiple processes, especially in oxidation stress, so we use the red square to circle it.

In the same disease network, deleting different nodes will cause different changes in the network structure and robustness. As is truly demonstrated in Figure 3, the total score of RI in disease (AMD and DR) subnetworks after HXMM and its constituent herbs attack can be easily seen. Higher scores indicate that the drug is more destructive to network stability and has a better potential efficacy, whereas the negative indicates that the drug is less destructive to the network. Using FDA-drugs as a positive control, the similarities and differences in the pathological mechanisms of AMD and DR can be discussed based on the results of Figure 3. On one hand, we found that HXMM had the highest total score of RI in the oxidative stress subnetwork both in AMD (21.63) and DR (19.62) (Figure 3A **)**. Then in AMD, total scores of RI in inflammation and angiogenesis were 11.15 (superior to 100% FDA-approved drugs), 7.77 (superior to 82.05% FDA-approved drugs). Three pathological processes of extracellular matrix, neuroprotection and blood coagulation demonstrated weaker ability to attack network robustness in AMD. On the other hand, in DR, the total scores of RI in angiogenesis, extracellular matrix, and inflammation were 14.90 (superior to 94.87% FDA-approved drugs), 5.29 (superior to 92.31% FDA-approved drugs), and 14.76 (superior to 87.18% FDA-approved drugs), respectively. Only the pathological process of blood coagulation demonstrated a weaker ability to attack network robustness in DR. Though they shared common pathological processes including oxidative stress, inflammatory response, blood coagulation, angiogenesis, and extracellular matrix, the effect and order of HXMMs regulating the major pathological processes of disease may be different. In addition to oxidative stress, inflammation and extracellular matrix may be more critical when HXMM attacks AMD, while angiogenesis, extracellular matrix, and inflammation may be more critical when attacked DR. In addition to blood coagulation, the results indicated that neuroprotection may not be the main process of HXMM in regulating AMD, and extracellular matrix may not be the main link of HXMM regulating DR. Thus, the key pathological processes of HXMM against retina injury may be oxidative stress, inflammation, and angiogenesis in Figure 3.

To confirm the advantages of HXMM and its constituent herbs in the treatment of diseases, a heatmap of HXMM and its herb for AMD total score of RI was depicted in Figure 3B. The total score of RI was corrected by FDA-approved drugs. In AMD, in the subnetwork of oxidative stress, HXMM had the highest total score of RI, followed by Conioselinum anthriscoides “Chuanxiong” (*Apiaceae*) (chuan xiong), *Curcuma aromatica* Salisb (Zingiberaceae) (yu jin,), *Angelica sinensis* (Oliv.) Diels (*Apiaceae*) (dang gui), *Salvia miltiorrhiza* Bunge (*Lamiaceae*) (dan shen), and *Typha domingensis* Pers (*Typhaceae*) (pu huang); in the subnetwork of inflammatory, the herbs with higher total scores of RI were Conioselinum anthriscoides “Chuanxiong” (*Apiaceae*) (chuan xiong), *Salvia miltiorrhiza* Bunge (*Lamiaceae*) (dan shen) and *Angelica sinensis* (Oliv.) Diels (*Apiaceae*) (dang gui); in the subnetwork of angiogenesis, *Angelica sinensis* (Oliv.) Diels (*Apiaceae*) (dang gui), Conioselinum anthriscoides “Chuanxiong” (*Apiaceae*) (chuan xiong), and *Curcuma aromatica* Salisb (*Zingiberaceae*) (yu jin) had certain advantages within all herbs (Figure 3B). Moreover, in DR, the subnetwork of oxidative stress in *Gentiana scabra* Bunge (*Gentianaceae*) (long dan), *Senna tora* (L.) Roxb (*Fabaceae*) (jue ming zi) and *Typha domingensis* Pers (*Typhaceae*) (pu huang) were with higher total score of RI; in the subnetwork of inflammatory, the herbs with higher total scores of RI were *Crataegus pinnatifida* Bunge (*Rosaceae*) (shanzha), *Angelica sinensis* (Oliv.) Diels (*Apiaceae*) (dang gui), Conioselinum anthriscoides “Chuanxiong” (*Apiaceae*) (chuan xiong), and *Typha domingensis* Pers (*Typhaceae*) (pu huang); and in the subnetwork of angiogenesis, Scutellaria baicalensis Georgi (*Lamiaceae*) (huang qin), *Typha domingensis* Pers (*Typhaceae*) (pu huang) and *Angelica sinensis* (Oliv.) Diels (*Apiaceae*) (dang gui) had certain advantages within all herbs (Figure 3C).

Further analysis showed that the whole HXMM was ahead of in this evaluation system, which meant higher total scores of RI were presented in most of the subnetworks, while several constituent herbs were exerted a better influence on the subnetworks, especially in oxidative stress, inflammation, and angiogenesis such as Conioselinum anthriscoides “Chuanxiong” (*Apiaceae*) (chuan xiong), *Typha domingensis* Pers (*Typhaceae*) (pu huang), *Angelica sinensis* (Oliv.) Diels (*Apiaceae*) (dang gui), *Salvia miltiorrhiza* Bunge (*Lamiaceae*) (dan shen) and so on.

This further implied that HXMM had quite a potential effect on AMD and DR in the regulation of the above processes.

### 3.5 He Xue Ming Mu Tablet Protects ARPE-19 Cells Against H_2_O_2_-Induced Retinal Injury Based on Anti-oxidation Activity

**FIGURE 4:**
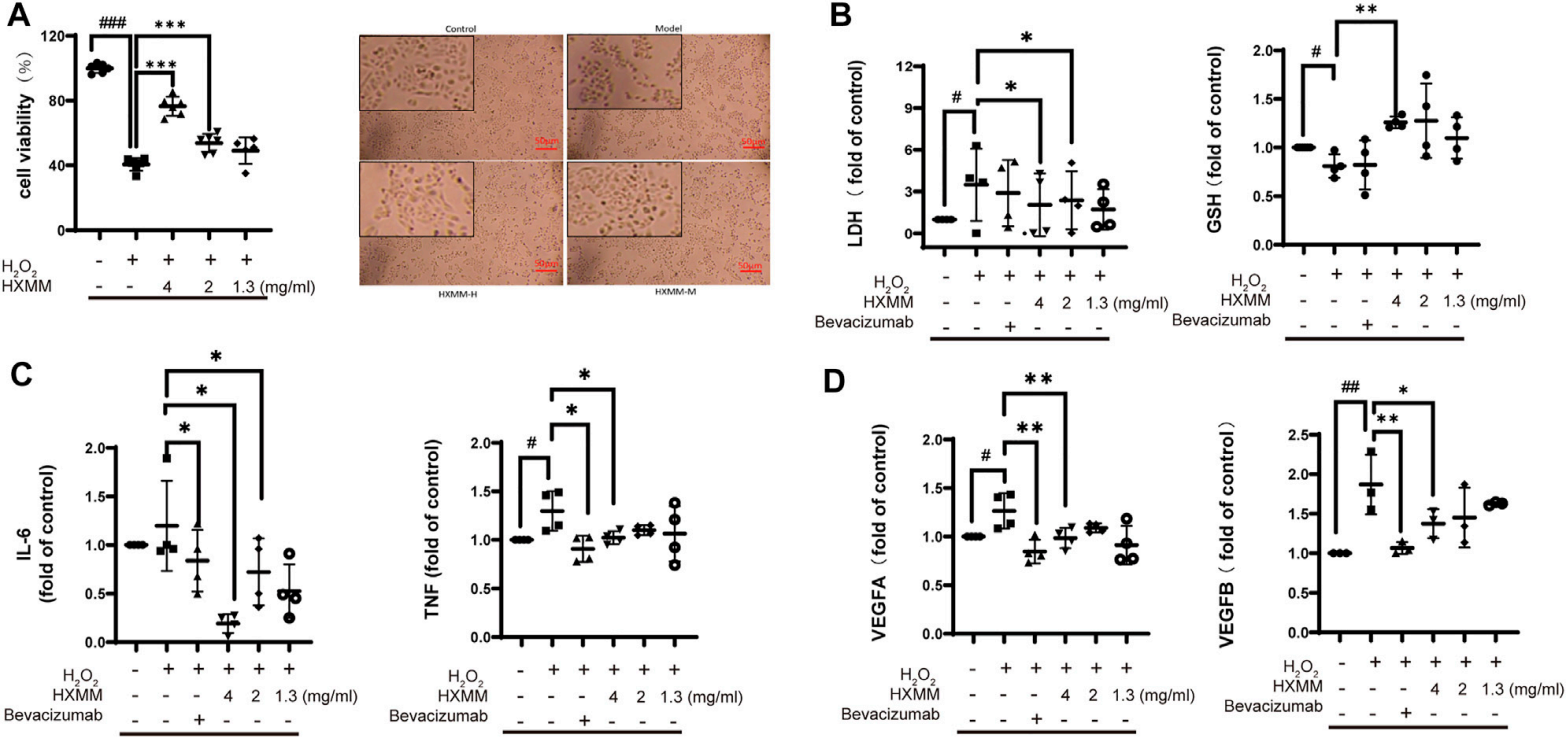
HXMM protects against H_2_O_2_-induced retinal injury in ARPE-19 cells **(A)** cell viability of viability of ARPE-19 cells treated with H_2_O_2_ and HXMM (n = 6), Changes in ARPE-19 cell morphology in response to HXMM and H_2_O_2_. APRE-19 cells without HXMM or H_2_O_2_ (control); APRE-19 cells with H_2_O_2_ (model); APRE-19 cells with 4 mg/ml and 2 mg/ml HXMM and then treated by 65 μM H_2_O_2_ (HXMM-H, HXMM-L) **(B)** fold change of process of oxidative stress, LDH activity (n = 4) and GSH level (n = 4) **(C)** fold change of process of inflammation, IL-6 activity (n = 4) and TNF-α level (n = 4) **(D)** fold change of process of angiogenesis, VEGFA level (n = 4) and VEGFB (n = 4). Data were expressed as the mean ± SD, #*p* < 0.05 versus control, *p < 0.05 versus model was considered significant, **p < 0.01 was considered significant statistical difference, ***p < 0.001 was considered extremely significant statistical difference.

In order to establish a retinal injury model in ARPE-19 cells, the CCK-8 assay was used to determine the proper concentration. With increasing concentrations of 50, 60, 80, and 100 μM for 12 h, the results showed that H_2_O_2_ decreased the cell growth rate, and around 65 μM was selected as IC_50_ concentration for subsequent study (Supplementary Figure S3A). Next, ARPE-19 cells were treated by concentrations of HXMM ranging from 0.5 to 16 mg/ml for 4 h to evaluate safe dosage, however, the concentrations of HXMM up to 16 mg/ml and 8 mg/ml did affect cell viability obviously (Supplementary Figure S3A). Therefore, we would better use lower than 4 mg/ml HXMM for the next part. The safe concentration of the positive drug was 0.5 mg/ml as indicated above (Supplementary Figure S3A). According to the cell viability of CCK-8, we found that 65 μm H_2_O_2_ significantly decreased the survival rate; both 4 mg/ml and 2 mg/ml HXMM can be effective to protect the injury of H_2_O_2_. Directly, more detailed morphologic changes in HXMM were as follows at the same condition. The ARPE-19 cells in control groups were mostly triangular or long spindle-shaped with dense and regular arrangement and visible nucleus. While the cell proliferation of model groups was inhibited and showed flattened shapes and irregular arrangement. Some could not even adhere to the wall tightly and was afloat in the culture medium. However, those pretreated in 4 and 2 mg/ml HXMM cells had less structural damage in nuclear pyknosis and fewer suspended cells (Figure 4A). To determine whether HXMM intervened in the process of oxidation stress, LDH, and GSH were measured in theARPE-19 cells. The activity of LDH was significantly increased followed by the administration of H_2_O_2_, which were significantly decreased pretreatment with high and middle dose (Figure 4B). GSH was expressed as a rising trend after HXMM attack. Moreover, several inflammatory-related proteins including IL-6 and TNF-α were significantly reduced in the model group, but HXMM at 4 mg/ml and 2 mg/ml significantly restored the activities of samples (Figure 4C). As an indicator of angiogenesis, the VEGFA and VEGFB enzyme were significantly increased in H_2_O_2_-treated cells. However, only the positive drug and HXMM in high dose reduced the activities relative to the model group (Figure 4D). These results confirmed that HXMM can elevate the GSH and decrease LDH levels to exhibit antioxidant activity, suppress the expression of IL-6, and TNF-α for anti-inflammatory activity, and decrease VEGFA and VEGFB levels to exhibit anti-angiogenesis activity.

### 3.6 Discovery of Potential Active Compound of He Xue Ming Mu Tablet Based on “Compound-Target-Pathological Process” Network

**FIGURE 5:**
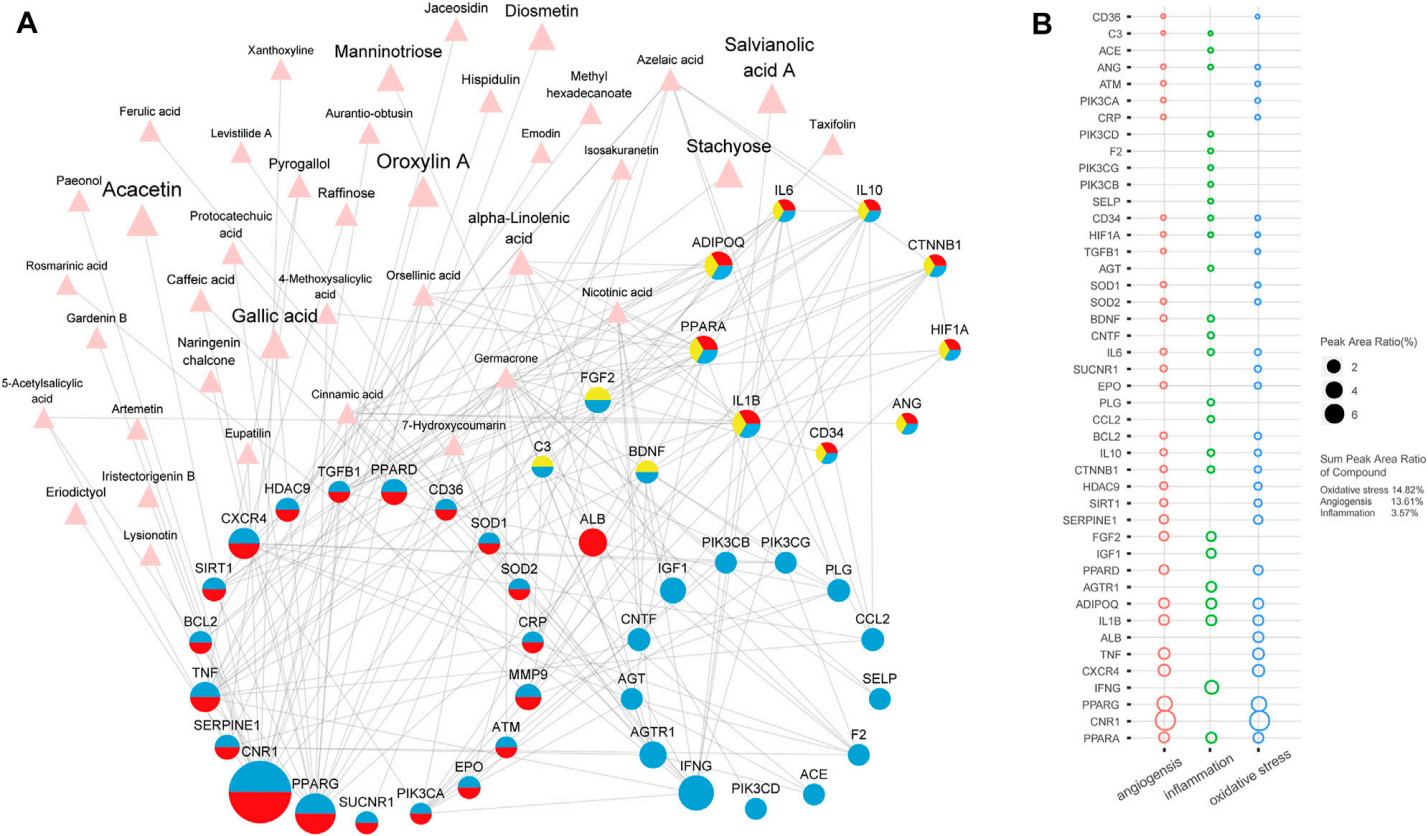
Analysis of the content ratio and action intensity in different pathological processes of HXMM. **(A)** Hierarchy network of chemical compound, drug-attacked nodes in the AMD network, and DR-related pathological processes. This network was composed of the interaction relationship between HXMM’s intervention in AMD and DR disease-related potential compounds and the targets of oxidative stress, inflammation, and angiogenesis. The pink triangle represents the potential compound of HXMM, the red circle represented the special target of oxidative stress, and the blue circle represented the special target of inflammation. A circle composed of multiple colors indicates that the target exists in multiple processes at the same time. There were 9 targets (composed of red, blue, and yellow) shared by oxidative stress, inflammation, and angiogenesis, 19 targets (composed of red and blue) can be seen in oxidative stress and inflammation, and 3 targets (composed of red and yellow) shared by oxidative stress and angiogenesis. **(B)** The bubble plot of the sum of peak area ratios of target-related compounds involved in the angiogenesis, inflammation, and oxidative stress. The abscissa denotes the three pathological processes mentioned in panel 5A, and the ordinate is the target in panel 5A. The size of the bubbles is related to the ratio of the total peak area of the target-related compounds.

To clarify the content ratio and action intensity in different pathological processes of the HXMM, a peak area ratio was introduced to describe the content ratio of the chemical compound. Also a new version of the “compound-target-pathological process” network was constructed, including common component compounds, potential targets, and related pathological interactions (Figure 5A). In Figure 5A, the size of the pink triangles is positively related to the peak area ratio of the compound, while the size of the targets is related to the sum of the peak area ratio of the compounds targeting it. In addition, the sum of the peak area ratio of all target-related compounds in the pathological processes was also calculated (Figure 5B). The sum ratio of the peak area ratio of oxidative stress-related compounds was 14.82%, angiogenesis was 13.61%, and inflammation was 3.56%. These results demonstrated that oxidative stress; angiogenesis are the two major pathological processes of HXMM against retinal degeneration disease.

Among them, Cannabinoid Receptor 1 (CNR1) was the target of 16 compounds, and the sums of the peak area ratio of 16 compounds were 7.38% (top of all targets in Figure 5A). Some studies confirmed that CNR1 played an important role in retinal injury (Rossi et al., 2013; Campbell et al., 2021). It is suggested that CNR1 and relevant signaling may promote de-differentiation and proliferation of Müller glia-derived progenitor cells. Peroxisome proliferator-activated receptors (PPARs) family is known to protect from retinal degenerations supported by recent studies, which is broadly expressed in all retinal cell types (Xu et al., 2020). Interferons (IFNs) are a group of signaling proteins synthesized and released by host cells in response to pathogens such as viruses, bacteria, or tumor cells. IFNG (IFN-γ), one of the major subtypes, is produced by leukocytes and is mainly involved in innate immunity in response to viral infection. It has been shown to be closely associated with retinal damage (Negishi et al., 2018; Alrashdi et al., 2019).

A total of 45 drug targets both in AMD and DR disease network were related to oxidative stress and inflammation and angiogenesis. There are 39 chemical compounds from HXMM in Figure 5A. Among them, 13 compounds were selected as potential active compounds of HXMM which had more than one disease target in this network. And 8/13 compounds were reported to have anti-oxidation activity, and 11/13 compounds had anti-inflammation activity, and 6/13 compounds had anti-VEGF activity (Table 1).

**TABLE 1:** Potential Compounds of HXMM Tablet by HPLC/MS.

Potential active compound of HXMM	Peak area ratio (%)	Herb	Anti-inflammation	Anti-VEGF	Anti-oxidation	
					ARPE wt H_2_O_2_	Other model
Germacrone	0.04	Yujin	An et al. (2014)	—	Chen et al. (2017)	Wu et al. (2019)
		Lü et al. (2ACT011)				
Nicotinic acid	0.16	Danggui	Digby et al. (2012)	Goldsborough (1960), Pan et al. (2017)	—	Kamat and Devasagayam (1999), Gambhir et al. (2012)
		Li et al. (2014)				
Pyrogallol	0.5	Chishao	Nicolis et al. (2008)	Santos et al. (2021)	Ozturk Sarikaya (2015)	—
		Liang et al. (2013)				
Caffeic acid	0.33	Danggui. Li et al. (2009)	Choi et al. (2018)	Jung et al. (2007)	—	Dinc et al. (2017), Paeng et al. (2015)
		Danshen. Jiang et al. (2005)				
		Chuanxiong. Wang et al. (2021)				
Protocatechuic acid	0.19	Danshen and Chuanxiong	Wang et al. (2015)	Hu et al. (2018)	Liu et al. (2002), Ya et al. (2021)	—
		Zhang et al. (2016)				
Cinnamic acid	0.02	Yujin. Yan et al. (2014)	Pontiki and Hadjipavlou-Litina (2018)	Choi et al. (2009), Zhang et al. (2019)	Taner et al. (2017), Li et al. (2011)	Hadjipavlou-Litina and Pontiki (2015)
		Danggui. Tao et al. (2017)				
alpha-Linolenic acid	0.79	Shanzha	Reifen et al. (2015)	Shen et al. (2012)	Zhu et al. (2020)	—
		Sun et al. (2008)				
Azelaic acid	0.19	Danggui	Akamatsu et al. (1991)	—	—	Passi et al. (1991)
		Chen et al. (2021a)				
7-Hydroxycoumarin	0.09	Xiakuca. Wu et al. (2020)	Timonen et al. (2011)	-	—	Kabeya et al. (2013)
		Danggui. Yang et al. (2019)				
Isosakuranetin	0.02		Manchope et al. (2017)	—	—	Wang et al. (2017), Chen et al. (2021)
Orsellinic acid	0.16	—	—	—	—	Zhu et al. (2020)
4-Methoxysalicylic acid	—	—	—	—	—	—
5-Acetylsalicylic acid	—	—	—	—	—	—

## 4 Discussion

The pathological changes of retinal degenerative diseases are irreversible. When patients have a cognitive impairment, the course of the disease is often in the middle or late stages. At this time, treatment can only slow down the development of the disease and cannot fundamentally reverse the damage of the retinal neural network. Therefore, early diagnosis and early treatment of such diseases should be done as far as possible to prevent the further development of the disease (Giridhar et al., 2002). Anti-VEGF therapy can be directly applied on vascular endothelial cells, inhibit their proliferation and migration speed and effectively inhibit VEGF activation, but intravitreal injection of these drugs is not “a single shot.”. Many countries recommend one injection per month. At present, a basic treatment plan is one injection per month and treatment according to the condition after 3 months. In addition, some patients will have problems such as bleeding at the injection site, increased intraocular pressure, conjunctival congestion, and so on. Therefore, seeking an effective method for the treatment of retinal degenerative damage is an important topic in the prevention and treatment of blindness (Gil-Martínez et al., 2020).

As mentioned in the introduction, the level of VEGFA should balance for cellular necessary baseline, which indicates the suppression of VEGF for anti-VEGF agents is not unconditionally beneficial. Also the majority of patients do not benefit from anti-VEGF therapy (Wang and Lo, 2018). Therefore, there is an urgent need for the development of new treatments (Wang and Lo, 2018). So anti-angiogenic therapy, anti-inflammatory therapy is necessary. HXMM provides an option for retinal degenerative patients for its retinal protection based on anti-oxidation, anti-inflammation, and anti-VEGF activity.

First of all, retinal degenerative diseases, e.g., AMD and DR, are complex with multiple factors, genes, and pathological processes; however, the internal causes of their pathogenesis are not the same. AMD is caused by long-term damage of ultraviolet rays, nutrition, metabolism, and genetic factors, which leads to the formation of choroidal neovascularization in the macular region of patients. DR is one of the complications of diabetes, directly related to the occurrence of diabetes. Poor blood sugar control state can lead to vascular lesions, fundus hemorrhage, exudation, severe macular edema, etc.

As shown in Figures 2A,B, they shared common pathological processes including oxidative stress, inflammatory response, blood coagulation, angiogenesis, and extracellular matrix. Although there are four main pathological processes in these two diseases, the number of specific targets is different. According to the number of targets from large to small, the pathological processes of AMD are inflammation, angiogenesis, oxidative stress, extracellular matrix, blood coagulation, and neuron death, while DR is inflammation, oxidative stress, angiogenesis, coagulation and extracellular matrix. And neuron death is a unique pathological process in AMD. Second, in Figure 3, compared to the clinical agents including anti-VEGF agents, growth factors, corticosteroids, and angiotensin II receptor blockers, immunesuppressants, anticoagulants, and antidiabetic drugs, we can apply the methods to the analysis of HXMM and its constituent herbs. The higher the ratio, the stronger is the potential efficacy. In the oxidative stress possess, the total score of HXMM is higher than that of FDA-approved drugs in AMD and DR, which indicates HXMM has stronger antioxidant activity than clinical agents. It was found that HXMM had a strong perturbation on the functional subnetwork for inflammation and blood coagulation in AMD and on angiogenesis and extracellular matrix in DR.

In addition, HXMM was predicted to have anti-inflammation and anti-angiogenesis effects similar to clinical agents. To validate the characteristics of HXMM, A retina injury cell model induced by H_2_O_2_ was designed to simulate the retinal injury process occurring in AMD and DR, and bevacizumab was used as a positive control, as commonly used clinical anti-VEGF drug. The results showed that HXMM and bevacizumab had good cytoprotective effects against H_2_O_2_-induced cell damage on APRE-19 cells. HXMM preferentially suppresses the activities of two oxidative indicators, LDH and GSH, HXMM and bevacizumab reduced the expression level of IL-6 and TNF-α to show their anti-inflammation activity. Angiogenesis, HXMM, and bevacizumab could modulate the expression of VEGFA to inhibit the occurrence of angiogenesis. And HXMM and bevacizumab both can suppress the activity of VEGFA. In summary, HXMM has a good intervention on the oxidative stress subnetwork compared to bevacizumab, while bevacizumab does have a stronger anti-VEGF ability.

In our study, overlay between pathological processes of AMD and DR shows that inflammation and angiogenesis have crosstalk based on their high overlap of composition targets. Also the common genes between oxidative stress and inflammation indicate the crosstalk of each other. The pathological process also has a unique composition, without the crosstalk with other processes. The network robustness under drug attack was used to quantitatively evaluate the drug intervention on the functional subnetwork for each pathological process. To cure AMD and DR, the most commonly used FDA-approved drugs like anti-VEGF agents in computational prediction shows higher robustness index to functional subnetwork, especially angiogenesis subnetwork, which is consistent with remarkable clinical benefits for anti-VEGF therapy. The robustness of inflammation and oxidative stress subnetwork also were reduced after anti-VEGF agent attack, which may be related to crosstalk between angiogenesis, inflammation and oxidative stress (Massengill et al., 2018). The strongest effect of anti-VEGF agents on reduction of network robustness implies that this approach can be used to evaluate and predict the effect of drugs on disease intervention.

Applying the methods to the analysis of HXMM and its constituent herbs, it was found that HXMM had strong perturbation on the functional subnetwork for oxidative stress, inflammation, and extracellular matrix in AMD, and on oxidative stress, extracellular matrix, and angiogenesis in DR. Comparing with the clinical agents, including anti-VEGF agents, growth factors, corticosteroids, angiotensin II receptor blockers, immunosuppressant drugs, anticoagulants and antidiabetic drugs, HXMM has stronger perturbation to network robustness of the oxidative stress subnetwork, which indicates HXMM has a stronger antioxidant activity than clinical agents. In addition to that, HXMM was predicted to have anti-inflammation and anti-angiogenesis effects similar to clinical agents. To validate the characteristics of HXMM, a retina injury cell model induced by H_2_O_2_ was designed to simulate the retinal injury process occurring in AMD and DR, and bevacizumab was used as a positive control, as a commonly used clinical anti-VEGF drug. The results showed that HXMM and bevacizumab had good cytoprotective effects against H_2_O_2_-induced cell damage on APRE-19 cells. HXMM preferentially suppresses the activities of two oxidative indicators, LDH and GSH, HXMM and bevacizumab reduced the expression level of IL-6 and TNF-α to show their anti-inflammation activity. Angiogenesis, HXMM, and bevacizumab could modulate the expression of VEGFA to inhibit the occurrence of angiogenesis. Also HXMM and bevacizumab both can suppress the activity of VEGFA. In summary, HXMM has a good intervention on the oxidative stress subnetwork compared to bevacizumab, while bevacizumab does have a stronger anti-VEGF ability.

To verify whether 13 potential compounds can be regarded as indicative compounds in HXMM after the drug attack in the pathology of oxidative stress, inflammation, and angiogenesis, based on the “compound-target-pathological process” network, here we conducted a round of preliminary literature mining. Germacrone is a sesquiterpenoid, often used as an indicator component of *Curcuma aromatica* Salisb (*Zingiberaceae*) (yu jin). This active compound has become a point of focus due to its rich pharmacological effects, such as anti-inflammatory, antioxidation, neuroprotective, and anticancer (Riaz et al., 2020). The reported data suggested that germacrone may show anti-inflammatory activity against H1N1 influenza and attenuate the injuries from cerebral reperfusion in rats by reducing MDA levels and elevating GSH and SOD activities (Liao et al., 2013; Wu et al., 2019). The anti-inflammatory therapeutic effect can be evaluated by regulating nicotinate and nicotinamide metabolism and arachidonic acid metabolism. Nicotinic acid can be found in many herbs of HXMM, such as *Chrysanthemum × morifolium* (Ramat.) Hemsl. (*Asteraceae*) (ju hua), *Angelica sinensis* (Oliv.) Diels (*Apiaceae*) (dang gui), Conioselinum anthriscoides “Chuanxiong” (*Apiaceae*) (chuan xiong) and *Typha domingensis* Pers (*Typhaceae*) (pu huang). Nicotinic acid belongs to the vitamin B family, which is an important coenzyme for the oxidoreduction reaction process. The reported data suggest that nicotinic acid may regulate arachidonic acid metabolism to improve the activities of acute inflammatory mice. Also nicotinic acid could inhibit the process of angiogenesis by changing the levels of TNF-α and VEGF to attenuate the severity of colitis (Ma Y. et al., 2016; Salem and Wadie, 2017). Cinnamic acid is also obtained from many natural products from HXMM, which is widely used for anti-inflammation and anti-VEGF. Moreover, cinnamic acid may have effects in the prevention and management of diabetes based on stimulation of insulin secretion and inhibition of protein glycation (Adisakwattana, 2017; Martins de Sá Müller et al., 2019). Shreds of evidence also demonstrate that other compounds in Table 1 could influence the pathology of inflammation, oxidative stress, and angiogenesis. In summary, those therapeutic compounds may serve as HXMM candidates in the near future. But there is a need for more strong evidence to be further explored and verified.

In addition, in order to make the overall experimental design more coherent, this study also conducted literature mining on the pharmacological effect of the relevant components of the monarch drugs in the HXMM formula on the H_2_O_2_-induced retinal injury model in ARPE-19 cells. Many ingredients found in HXMM intestinal absorption liquid have been reported to exert certain pharmacological effects in this model, such as salvianolic acid A in *Salvia miltiorrhiza* Bunge (*Lamiaceae*) (dan shen), isorhamnetin in *Typha domingensis* Pers (*Typhaceae*) (pu huang), salidroside in *Ligustrum lucidum* W.T. Aiton (*Oleaceae*) (nu zhen zi), paeoniflorin in *Paeonia lactiflora* Pall (*Paeoniaceae*) (chi shao) and ferulic acid in *Angelica sinensis* (Oliv) Diels (*Apiaceae*) (dang gui), and Ligusticum chuanxiong (chuan xiong). Action intensity for drug-targeted interaction should also be considered in the analysis. This information about the relative amounts of compounds (i.e. peak areas) could conceivably lead to help us discover more valuable disease targets and potentially active compounds. At the same time, an important matter to the strength of the drug and the target to resolve for future research. And on a broader level, research is also encouraged to be validated by animal experiments for further investigation.

## Data Availability

The raw data supporting the conclusions of this article will be made available by the authors, without undue reservation.
