# Supplementary material for: Exploration of the Specific Pathology of HXMM Tablet Against Retinal Injury Based on Drug Attack Model to Network Robustness
Source: Front Pharmacol. 2022 Mar 25;13:826535. doi: 10.3389/fphar.2022.826535 (PMC8990835; doi:10.3389/fphar.2022.826535)
Supplement: Supplementary file 1 [file Presentation1.pdf]

Supplementary Material

Table S1. Information about the compounds of HXMM Intestinal Absorption Solution

Name	Formula	Annot. DeltaMass [ppm]	Calc. MW	RT [min]	Group Area: HE-Y-NEG	Group Area: HE-Y-POS
Baicalein	C15 H10 O5	-1.17	270.0525	28.625	31292367060	35223271980
Baicalin	C21 H18 O11	-0.9	446.0845	24.092	25396200260	35042695887
Wogonin	C16 H12 O5	-0.71	284.0683	32.15	16488475452	32655955916
Gentiopicrin	C16 H20 O9	-1.12	356.1103	20.044	14975773983	982674741.5
Wogonoside	C22 H20 O11	-0.77	460.1002	26.01	14345987949	17033289956
Citric acid	C6 H8 O7	-0.42	192.0269	3.524	12415622458	897955873.3
3,5-Dicaffeoylquinic acid	C25 H24 O12	-500001	258.063	22.726	7580485810	
Paeoniflorin	C23 H28 O11	-0.96	480.1627	20.902	6503462924	
Specnuezhenide	C31 H42 O17	-0.69	686.2417	22.16	5841670262	1949915919
Oroxylin A-7-O-β-D-	C22 H20 O11	-0.81	460.100	25.42	571808373	765278052

glucuronide			2	8	1	7
Danshensu	C9 H10 O5	-0.96	198.0526	17.259	5384917758	
Acacetin	C16 H12 O5	-0.85	284.0682	32.632	5079888540	4527043590
Chrysosplenetin B	C19 H18 O8	-0.57	374.1	32.568	4787737187	12419541500
Oroxylin A	C16 H12 O5	-0.63	284.0683	32.951	4737550053	15218975072
Apigenin	C15 H10 O5	-1.19	270.0525	27.663	4627635872	3506769582
Isoacteoside	C29 H36 O15	-0.84	624.2049	21.75	4110807172	411517919
Salvianolic acid A	C26 H22 O10	-0.7	494.121	24.86	4063789424	182760828.9
Luteolin	C15 H10 O6	-1	286.0475	25.743	3909438436	2339305111
Gallic acid	C7 H6 O5	-0.89	170.0214	8.148	3870081301	273010668
Protocatechualdehyde	C7 H6 O3	-1.35	138.0315	18.673	3581569487	290979563.3
Stachyose	C24 H42 O21	-0.09	666.2218	2.437	3561042395	
1-Caffeoylquinic acid	C16 H18 O9	-0.64	354.0949	19.362	3396787405	545533070.6

Sucrose	C12 H22 O11	-0.87	342.1159	2.089	3210427555	
Geniposidic acid	C16 H22 O10	-0.78	374.121	17.764	3200341647	121594143.4
Manninotriose	C18 H32 O16	-0.31	504.1689	2.688	3121912506	
Diosmetin	C16 H12 O6	-1.03	300.0631	28.371	2963920927	4089793919
Isochlorogenic acid C	C25 H24 O12	-1.27	516.1261	23.125	2825741668	403226922.5
Loganic acid	C16 H24 O10	-0.71	376.1367	18.47	2722191559	113216973.6
Chrysin	C15 H10 O4	-0.93	254.0577	32.341	2591635774	1838249819
Lithospermic acid	C27 H22 O12	-0.96	538.1106	21.757	2381195350	
Albiflorin	C23 H28 O11	-0.63	480.1629	23.251	2302964728	1394503569
Scutellarein	C15 H10 O6	-0.98	286.0475	24.492	2174645928	277676044.8
Quercitrin	C21 H20 O11	-0.49	448.1003	24.688	2031368672	748804100.7
Apigenin-7-O-β-D-glucoside	C21 H20 O10	-0.61	432.1054	23.01	1862052691	1979827206
Narcissoside	C28 H32 O16	-0.48	624.168	21.88	176606296	675784192.

			7	7	2	2
Typhaneoside	C34 H42 O20	-0.67	770.2264	21.217	1704290242	743920024.5
Ecliptasaponin A	C36 H58 O9	-0.57	634.4077	32.334	1587768565	
Verbascoside	C29 H36 O15	-0.76	624.205	22.267	1419426871	106434211.9
Benzoylpaeoniflorin	C30 H32 O12	-0.11	584.1893	26.748	1324947725	572987181.5
Pyrogallol	C6 H6 O3	-1.42	126.0315	8.147	1293897807	
Jaceosidin	C17 H14 O7	-0.65	330.0737	30.435	1099815849	846666017.9
Raffinose	C18 H32 O16	91249.86	550.1744	1.659	995473376.4	
Eriodictyol	C15 H12 O6	-1.02	288.0631	25.409	966948693.6	396065762.7
Quinic acid	C7 H12 O6	-0.74	192.0633	19.355	952329819.4	
Baicalin methyl ester	C22 H20 O11	-0.81	460.1002	26.314	945667103.9	1441994255
Caffeic acid	C9 H8 O4	-0.9	180.0421	20.063	848704789.5	
Naringenin chalcone	C15 H12 O5	-0.64	272.0683	27.468	848209353.8	330352132.2

Astilbin	C21 H22 O11	-0.8	450.1159	21.845	775195351	146663556
Paeonolide	C20 H28 O12	-0.29	460.1579	20.213	775129034.5	
Linarin	C28 H32 O14	-0.35	592.179	24.85	772122065.6	2198220043
Oxypaeoniflorin	C23 H28 O12	-0.06	496.1581	19.162	653600357.8	
Diosmetin-7-O-β-D-glucopyranoside	C22 H22 O11	-0.85	462.1158	23.41	645999081.8	1552290531
Pectolinarigenin	C17 H14 O6	-1.11	314.0787	32.514	634898481.2	2778990605
Salidroside	C14 H20 O7	-0.97	300.1206	18.509	595860396	187714910.6
Hesperetin	C16 H14 O6	-1.46	302.0786	28.171	591997639.5	288385455.1
Cryptochlorogenic acid	C16 H18 O9	-0.69	354.0948	18.268	590462411.5	
Aurantio-obtusin β-D-glucoside	C23 H24 O12	-0.3	492.1266	23.827	576113646.7	244218027.9
Apigenin 7-O-glucuronide	C21 H18 O11	-1.43	446.0843	21.463	545649411.8	
Iristectorigenin B	C17 H14 O7	-0.59	330.0738	28.929	510373624	749596486
Protocatechuic acid	C7 H6 O4	-1.28	154.026	19.96	486629844.	

			4	6	2	
Azelaic acid	C9 H16 O4	-0.97	188.104<br>7	23.39<br>6	485938433.<br>8	13794187.7<br>8
Luteolin 7-<br>glucuronide	C21 H18 O12	-0.73	462.079<br>5	22.07<br>4	475154723.<br>4	666665203.<br>8
Taxifolin	C15 H12 O7	-0.9	304.058	21.19<br>5	466950076.<br>2	86134416.0<br>5
Methyl<br>hexadecanoate	C17 H34 O2	170227.<br>8	316.260<br>9	38.17	448742937.<br>5	
Rutin	C27 H30 O16	-0.7	610.153	21.57<br>3	433497139.<br>5	185781634.<br>8
Orsellinic acid	C8 H8 O4	-0.78	168.042<br>1	19.74<br>6	417616472.<br>8	
Kaempferol 3-<br>glucorhamnoside	C27 H30 O15	-0.41	594.158<br>2	21.79<br>6	416705474.<br>3	155405897.<br>6
Diosmin	C28 H32 O15	-0.08	608.174<br>1	22.83<br>9	411525228.<br>2	853677695.<br>3
Lysionotin	C18 H16 O7	-0.57	344.089<br>4	31.72	393918633	908592922.<br>6
Wedelolactone	C16 H10 O7	-0.79	314.042<br>4	26.17<br>4	392995537.<br>6	146004950.<br>1
Hastatoside 5-	C17 H24 O11	113837.<br>4	450.137<br>2	18.84<br>2	383234859.<br>9	
Nystose	C24 H42 O21	69054.3<br>4	712.227<br>4	1.614	378074167.<br>3	

Scutellarin methyl ester	C22 H20 O12	-96631.6	430.0896	23.465	317465015.5	
Quercetin-3-O-β-D-glucose-7-O-β-D-gentiobioside	C33 H40 O22	-0.73	788.2006	19.075	304793128.1	170901896.1
p-Hydroxybenzaldehyde	C7 H6 O2	-0.91	122.0367	20.402	290272233	45099433.15
Mannitol	C6 H14 O6	-0.43	182.079	2.32	239252170.4	
Forsythoside E	C20 H30 O12	-0.29	462.1736	18.174	232437933.4	28140953.76
7-Hydroxycoumarin	C9 H6 O3	-1.9	162.0314	23.38	231437068.4	
3,4-Dihydroxyphenylethanol	C8 H10 O3	-1.02	154.0628	17.529	174566164.6	
Puerarin	C21 H20 O9	-0.39	416.1106	22.913	147681338.1	189867886.5
Purpureaside C	C35 H46 O20	-0.53	786.2578	20.077	146878802.4	
Ellagic acid	C14 H6 O8	-1.2	302.0059	21.916	123254011.2	
Salvianolic acid C	C26 H20 O10	2.52	492.1069	25.802	112241425.1	
Rhoifolin	C27 H30 O14	0.06	578.1636	23.601	111492290.8	8159898.937

Maltopentaose	C30 H52 O26	-0.31	828.2744	2.609	108094897.2	
1,3-Dicaffeoylquinic acid	C25 H24 O12	-0.67	516.1264	20.446	94700595.59	23995767.06
6''-O-Acetylglycitin	C24 H24 O11	-0.05	488.1318	29.037	83477507.2	174563795.6
Vanillin	C8 H8 O3	-0.77	152.0472	19.785	75635165.47	10759996.12
Hyperoside	C21 H20 O12	-0.53	464.0952	21.103	69324128.07	
Plantamajoside	C29 H36 O16	-0.58	640.2	21.223	67777600.28	
Pinocembrin	C15 H12 O4	-0.67	256.0734	32.732	60299337.72	21521899.65
Sibiricose A5	C22 H30 O14	-0.9	518.1631	20.114	57945971.18	
4-Methoxysalicylic acid	C8 H8 O4	-0.78	168.0421	17.624	56812471.51	
Tectorigenin	C16 H12 O6	-0.93	300.0631	25.103	48725181.48	134064551.2
Demethylwedelolactone	C15 H8 O7	-1.36	300.0266	22.226	43719005.37	
Irigenin	C18 H16 O8	-0.83	360.0842	28.564	43549941.54	249812306.7

7,8-Dihydroxycoumarin	C9 H6 O4	-1.37	178.0264	21.745	42604229.38	
Casticin	C19 H18 O8	-0.8	374.0999	31.034	41333243.31	
Aucubin	C15 H22 O9	0.31	346.1265	17.144	40185718.49	
Kaempferol	C15 H10 O6	-1.17	286.0474	22.053	38734659.55	206636648.3
Spiculisporic acid	C17 H28 O6	-0.58	328.1884	36.232	37474022.4	
Cichoric acid	C22 H18 O12	-1.11	474.0793	21.863	34010897.84	
Shanzhiside	C16 H24 O11	-0.37	392.1317	17.386	31405592.28	
Glomeratose A	C24 H34 O15	-1.52	562.1889	19.113	28824966.27	
2''-O-β-L-Galactopyranosylorientin	C27 H30 O16	-0.8	610.1529	18.601	27062294.77	141118913.5
Calceolarioside B	C23 H26 O11	-1.18	478.147	21.762	24866302.48	69518037.42
p-Coumaric acid	C9 H8 O3	-1.01	164.0472	19.287	21930236.71	
Eriocitrin	C27 H32 O15	0.75	596.1746	21.401	20784279.21	6482891.283

5-Acetylsalicylic acid	C9 H8 O4	116.74	180.0633	19.42	20119255.36	
Columbianetin acetate	C16 H16 O5	-0.7	288.0996	21.078	17533432.01	13646169.7
Gardenoside	C17 H24 O11	-0.63	404.1316	19.115	16795785.15	
Asperulosidic acid	C18 H24 O12	-0.97	432.1264	17.683	16273042.49	
Pedunculoside	C36 H58 O10	70733.87	696.4085	28.456	8830054.858	
Isosakuranetin	C16 H14 O5	-0.85	286.0839	25.595	8789192.232	39907908.67
Quercetin 3-O-β-D-Glucuronide	C21 H18 O13	0.28	478.0749	21.075	8659611.538	8567864.684
Naringin dihydrochalcone	C27 H34 O14	-0.45	582.1946	21.598	7144876.056	
Purpurin	C14 H8 O5	-0.41	256.0371	24.794	6773169.262	15466781.6
4',7-Di-O-methylnaringenin	C17 H16 O5	-2.03	300.0992	27.956	5831158.053	
Isosakuranin	C22 H24 O10	-133933	388.1164	25.204	4322368.935	
Linderalactone	C15 H16 O3	-0.58	244.1098	32.709		33732451.83

Sweroside	C16 H22 O9	-0.5	358.1262	18.493	33343890.96
Demethoxyyangonin	C14 H12 O3	-0.09	228.0786	20.994	17962946.84
5-Hydroxy-1-tetralone	C10 H10 O2	-0.44	162.068	18.896	10888510.03
Homoorientin	C21 H20 O11	0.43	448.1008	20.864	26343644.27
Isopimpinellin	C13 H10 O5	-0.23	246.0528	30.022	15759989.63
Abscisic acid	C15 H20 O4	-0.13	264.1361	20.466	19709372.97
Glabrolide	C30 H44 O4	0.62	468.3243	33.349	20780963.18
Isosinensetin	C20 H20 O7	0.15	372.121	29.268	13027248.18
Xanthotoxol	C11 H6 O4	-0.28	202.0266	22.981	22057023.34
Isofraxidin	C11 H10 O5	-1.15	222.0526	22.507	22072682.42
Xanthoxyline	C10 H12 O4	-0.59	196.0734	18.493	106971880.3
Isoeugenol acetate	C12 H14 O3	-0.85	206.0941	24.053	329956593.6
Eupatilin	C18 H16 O7	-0.4	344.089	31.22	312585002.

			5	4	5
5-Hydroxymethylfurfural	C6 H6 O3	0.06	126.0317	2.046	307178816.5
Adenine	C5 H5 N5	0.44	135.0546	1.642	267101172.5
4-Methylumbelliferone	C10 H8 O3	-0.42	176.0473	17.778	243072840.8
5,7,3'-Trihydroxy-6,4',5'-trimethoxyflavone	C18 H16 O8	-0.84	360.0842	28.29	234123381.3
3-Butylidenephthalide	C12 H12 O2	0.23	188.0838	29.633	219047278.5
Ginkgolic Acid C15:1	C22 H34 O3	-0.2	346.2507	48.313	197533247.7
Arglabin	C15 H18 O3	-0.24	246.1255	34.873	167397516
Nicotinic acid	C6 H5 N O2	0.51	123.0321	2.888	354857458.5
Paeonol	C9 H10 O3	-0.05	166.063	17.777	384603084.3
Stachydrine	C7 H13 N O2	0.26	143.0947	1.637	3922208519
Dihydrotanshinone I	C18 H14 O3	-0.07	278.0943	36.759	3034918394

α-Linolenic acid	C18 H30 O2	-0.28	278.2245	40.426	1784033478
Hispidulin	C16 H12 O6	-0.77	300.0632	29.256	871490764.3
Cantharidin	C10 H12 O4	-0.52	196.0735	23.258	689049564.2
Ferulaldehyde	C10 H10 O3	-0.23	178.063	20.918	619807575.1
Skimmin	C15 H16 O8	-1.56	324.084	21.763	609761669.5
Wilforlide A	C30 H46 O3	-0.37	454.3445	32.337	590879065.2
Astragalin	C21 H20 O11	-0.66	448.1003	18.606	513132604.1
Cinnamaldehyde	C9 H8 O	-0.6	132.0574	23.259	162455788.7
Iridin	C24 H26 O13	0.42	522.1376	23.175	120873168.4
Coptisine chloride			319.0844	23.773	49307841.1
Curcumenol	C15 H22 O2	0.1	234.162	33.759	48708353.23
Curcumol	C15 H24 O2	0.06	236.1776	36.005	48478538.92
Anisic aldehyde	C8 H8 O2	-0.1	136.052	21.68	47255489.9

			4	4	6
Cinnamic acid	C9 H8 O2	-0.2	148.0524	19.221	54832745.44
Senkyunolide A	C12 H16 O2	-0.12	192.115	33.987	42411738.6
3,5-Dimethoxy-4-hydroxybenzaldehyde	C9 H10 O4	-0.25	182.0579	23.39	40700093.45
Sinensetin	C20 H20 O7	0.07	372.1209	30.829	39646224.48
5-O-Demethylnobiletin	C20 H20 O8	-0.28	388.1157	29.546	38284683.48
Coumarin	C9 H6 O2	-0.36	146.0367	21.485	55540143.78
Quercetin	C15 H10 O7	-1.32	302.0423	21.592	62923468.84
4-Methyl-6,7-dihydroxycoumarin	C10 H8 O4	-1.17	192.042	22.167	115430194.1
18 β-Glycyrrhetintic Acid	C30 H46 O4	38292.26	488.35	26.973	108661612.9
Decursinol	C14 H14 O4	-0.11	246.0892	30.509	92678030.52
Germacrone	C15 H22 O	-0.05	218.1671	34.026	79462701.47
Isoquercitrin	C21 H20 O12	0.07	464.0955	19.092	75256235.7

α-Cyperone	C15 H22 O	82553.73	236.1776	27.58	71160496.31
L-Phenylalanine	C9 H11 N O2	0.48	165.0791	20.162	67135672.74
2-Hydroxy-4-methoxybenzaldehyde	C8 H8 O3	-0.66	152.0472	21.538	67057122.39
Demethyleneberberine	C19 H17 N O4	-0.53	323.1156	22.576	125950784
Cafestol	C20 H28 O3	-0.9	316.2036	25.861	2244172.747

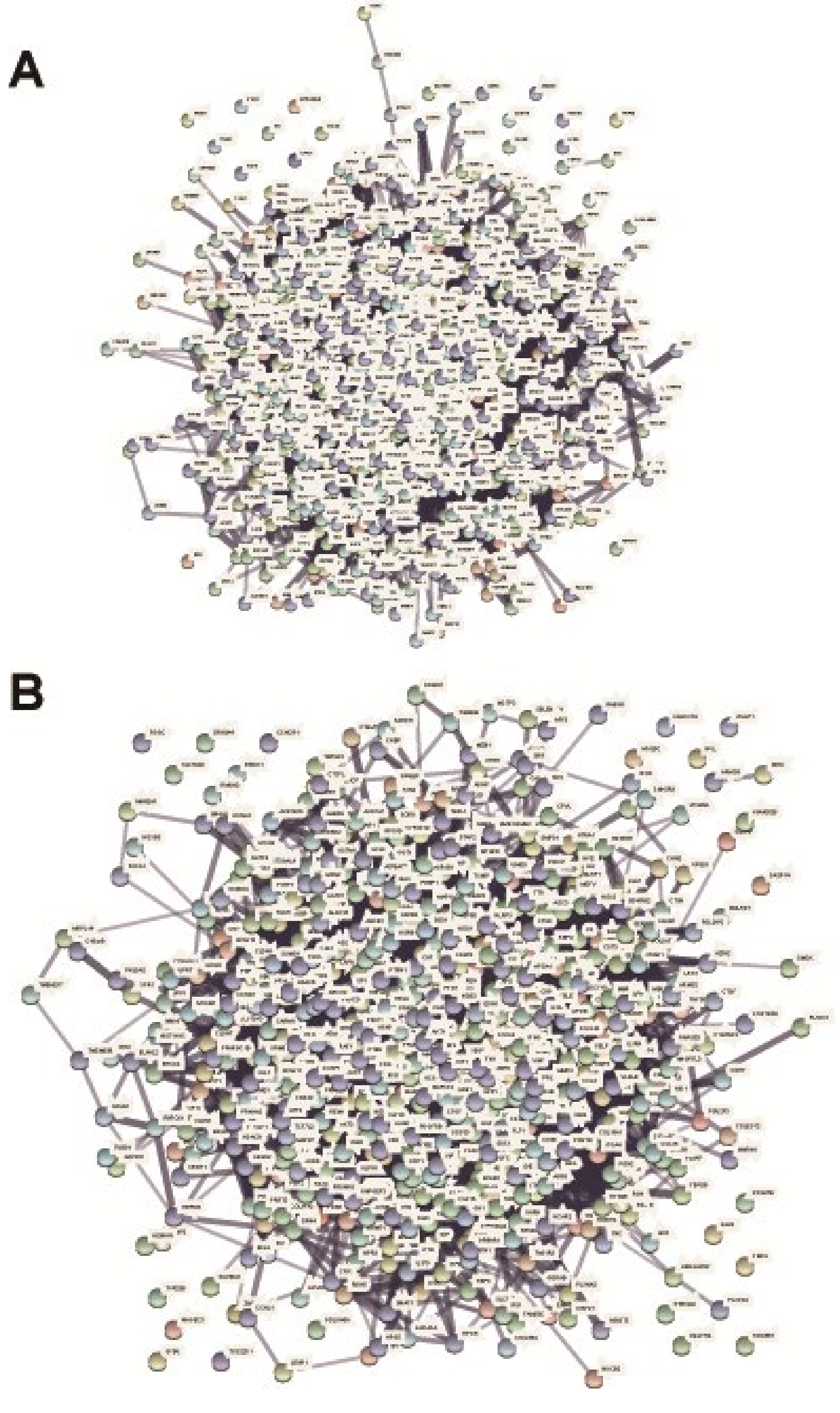
A
B

Supplementary Figure 1. The information about disease network

(A)protein protein interaction network of AMD

(B)protein protein interaction network of AMD

A

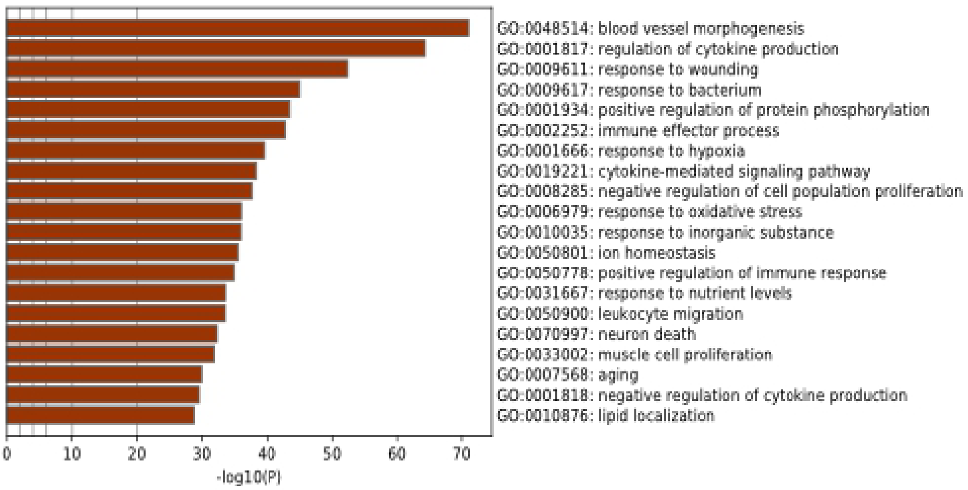

B

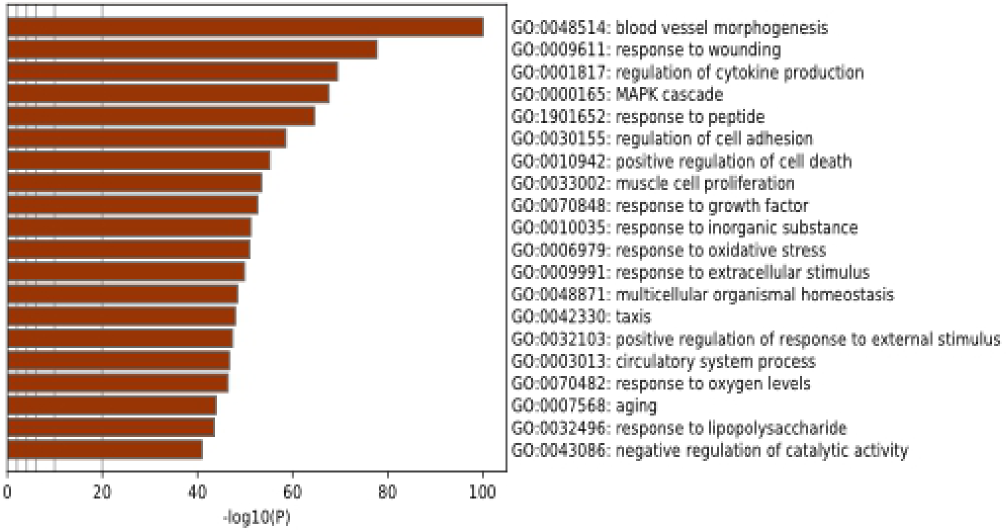

Supplementary Figure 2. Functional enrichment analysis of GO biological process of different kinds of diseases

(A) Functional enrichment analysis of GO biological process of different kinds of AMD

(B) Functional enrichment analysis of GO biological process of different kinds of DR

The result of GO biological processes can be viewed by terms across input gene lists with a p-value < 0.01, a minimum count of 3

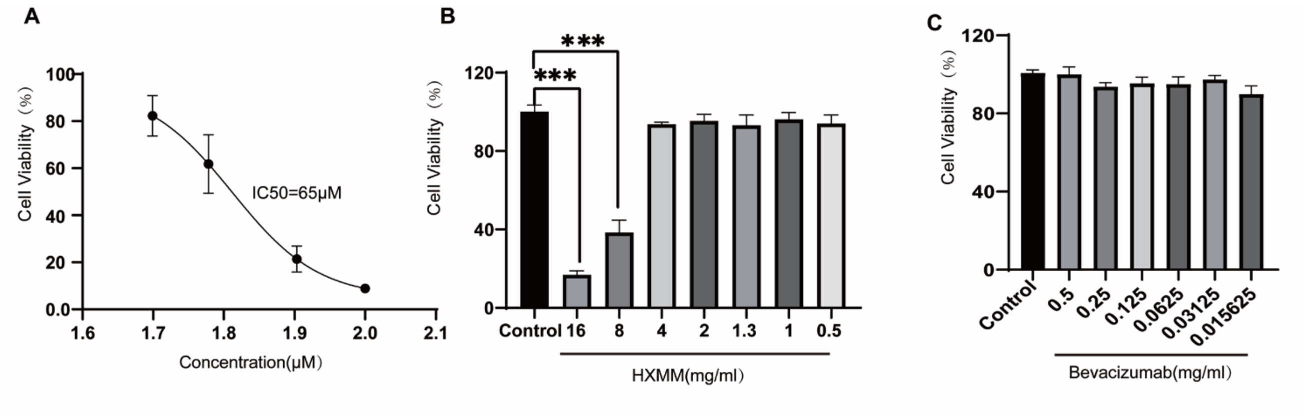

Supplementary Figure 3. The cell viability of drug attack cell

(A) Determination of the concentration of H_2O_2 (n = 3)
(B) Determination of the concentrations of HXMM (n = 3)
(C) Determination of the concentrations of positive control (n = 3)
